# Design of a robotic device for the delivery of transcranial, magnetic resonance guided focused ultrasound for intraventricular hemorrhage of prematurity

**DOI:** 10.1186/2050-5736-3-S1-P32

**Published:** 2015-06-30

**Authors:** Karl Price, Vivian Sin, Charles Mougenot, Thomas Looi, Samuel Pichardo, Adam Waspe, James Drake

**Affiliations:** 1 Centre for Image Guided Innovation and Therapeutic Intervention, Toronto, Canada; 2 Philips Healthcare Canada, Toronto, Canada; 3 Thunder Bay Research Institute/Lakehead University, Toronto, Canada

## Background/introduction

Premature birth affects 12.5% of pregnancies, and as a result, intraventricular hemorrhage (IVH) of the brain with subsequent development of hydrocephalus is a major cause of morbidity, mortality, and poor intellectual outcomes. Prospective clinical trials to dissolve IVH clots with intraventricular infusion of tissue plasminogen activator through surgically implanted catheters demonstrated improved intellectual outcome in the survivors but at an increased risk of hemorrhage. A non-invasive method to lyse the clots may result in a reduced risk of subsequent hydrocephalus, and better intellectual outcome for these patients. Magnetic resonance guided focused ultrasound (MRgFUS) delivered through the open fontanel is such a therapy, but requires a versatile transducer positioning system that adapts to the MRI compatible transport and imaging incubator used to manage these fragile neonates.

## Methods

A five degree of freedom MRI compatible robotic device has been designed to precisely position an MRgFUS transducer (Philips Sonalleve brand) for transcranial therapies within the constraints of the neonatal incubator system. Pulsed focused ultrasound (FUS) energy is used to lyse IVH blood clots in the brain of the patient. The robot is used to position the transducer above the head of the patient while the patient is inside the MRI machine. Five ultrasonic, non-magnetic motors are used to actuate the robot. Specially selected, non-magnetic materials are used to construct the robot. A workspace analysis for delivery of the FUS to the intraventricular system of the brain in typical premature neonates was carried out, as were predicted load and speed requirements. A mock-up of the treatment system, with a 3D printed neonatal skull inside the incubator was constructed. The first prototype of the robot was tested for MRI compatibility while the motors were in operation. A master-slave control system, integrated with the MRI imaging system was also designed.

## Results and conclusions

Tests of individual components of the robot show that it has the potential for highly accurate targeting with the FUS transducer through the anterior fontanel of a neonatal head within the incubator transport system in the MRI environment.

The design meets workspace, load and speed requirements. The activated components do not create significant imaging artifacts or degradation of signal to noise ratio (SNR). Geometric distortions due to large metal objects (such as the drive motors) are negligible at distances longer than half the focal length of the transducer. We conclude from these experiments that the design of the robot is appropriate for transcranial MRgFUS thrombolysis and could significantly improve treatment of IVH in premature infants.

